# Relation of Mean Platelet Volume (MPV) with Cancer: A Systematic Review with a Focus on Disease Outcome on Twelve Types of Cancer

**DOI:** 10.3390/curroncol30030258

**Published:** 2023-03-14

**Authors:** Paraskevi Detopoulou, George I. Panoutsopoulos, Marina Mantoglou, Periklis Michailidis, Ifigenia Pantazi, Spyros Papadopoulos, Andrea Paola Rojas Gil

**Affiliations:** 1 Department of Clinical Nutrition, General Hospital Korgialenio Benakio, Athanassaki 2, 11526 Athens, Greece; 2 Department of Nutritional Science and Dietetics, Faculty of Health Sciences, University of Peloponnese, New Building, Antikalamos, 24100 Kalamata, Greece; 3 Laboratory of Basic Health Sciences, Department of Nursing, Faculty of Health Sciences, University of Peloponnese, 22100 Tripoli, Greece

**Keywords:** mean platelet volume, MPV, cancer, survival, inflammation

## Abstract

Inflammatory proteins activate platelets, which have been observed to be directly related to cancer progression and development. The aim of this systematic review is to investigate the possible association between Mean Platelet Volume (MPV) and cancer (diagnostic capacity of MPV, relation to survival, the severity of the disease, and metastasis). A literature review was performed in the online database PubMed and Google Scholar for the period of 2010–2022. In total, 83 studies including 21,034 participants with 12 different types of cancer (i.e., gastric cancer, colon cancer, esophageal squamous cell carcinoma, renal cancer, breast cancer, ovarian cancer, endometrial cancer, thyroid cancer, lung cancer, bladder cancer, gallbladder cancer, and multiple myeloma) were identified. The role of MPV has been extensively investigated in several types of cancer, such as gastric, colon, breast, and lung cancer, while few data exist for other types, such as renal, gallbladder cancer, and multiple myeloma. Most studies in gastric, breast, endometrium, thyroid, and lung cancer documented an elevated MPV in cancer patients. Data were less clear-cut for esophageal, ovarian, and colon cancer, while reduced MPV was observed in renal cell carcinoma and gallbladder cancer. Several studies on colon cancer (4 out of 6) and fewer on lung cancer (4 out of 10) indicated an unfavorable role of increased MPV regarding mortality. As far as other cancer types are concerned, fewer studies were conducted. MPV can be used as a potential biomarker in cancer diagnosis and could be a useful tool for the optimization of treatment strategies. Possible underlying mechanisms between cancer and MPV are discussed. However, further studies are needed to elucidate the exact role of MPV in cancer progression and metastasis.

## 1. Introduction

Cancer is an emerging epidemic with economic, social, and psychosomatic effects [1]. It is a life-threatening disease characterized by abnormal cell growth and division [1]. Most cancers are curable when diagnosed early and treatment begins soon after diagnosis [1]. To develop appropriate treatment strategies against cancer, research into easily detectable and accessible biomarkers is required [2]. Platelet-based markers are potential candidates for cancer diagnosis and monitoring, given the emerging role of platelets in cancer biology [3,4].

Platelets are the smallest but highly active morphological components of blood [5]. They are produced by the megakaryocytes of the bone marrow and under normal conditions are 157.000–351.000 in women and 135.000–317.000 in men per microliter of blood [5]. The average lifespan of platelets is 5–9 days [5]. They play a major role in the coagulation process and also participate in fibrosis, normal hemostasis, and other pathophysiological processes [5]. Platelets accumulate at the site of damage, and changes in their morphology are observed upon their activation with inflammatory markers [6] and several agonists such as Platelet-Activating Factor (PAF) and Adenosine Diphosphate (ADP) in vitro and ex vivo [7,8]. The number of platelets is determined by the balance between the rate of production and consumption and genetic factors [5]. Platelets differ in functional activity and size, and the function of platelets is related to their size [5]. New and more active platelets are larger than old ones. In other words, larger platelets may be younger and more metabolically and enzymatically active than smaller ones, they aggregate more easily and could be more easily stimulated to release chemical mediators [9]. This suggests that platelet volume reflects platelet activation. Activated platelets play an important role during the formation and development of clots [10]. They are active in systemic inflammation and have a higher prothrombotic potential in health and disease [8,11].

Tumor cells secrete cytokines that contribute to a prothrombotic microenvironment, which includes platelet activation [4]. By secreting proinflammatory and growth factors, platelets play an important role in cancer progression and metastasis, since inflammation is a critical component of tumor progression [4]. Complicated interactions between platelets and cancer cells lead to tumor growth, neoangiogenesis, tumor cell dissemination, the release of adhesion molecules, and growth factors, all of which provide basic ingredients for tumor growth and metastasis [4].

Mean Platelet Volume (MPV) is one of the key platelet parameters, along with platelet count (PLT) and Platelet Distribution Width (PDW). MPV is a non-invasive, low-cost parameter, easily assessed and readily available in clinical practice, which shows the average size of platelets in the bloodstream and reflects their production rate and their degree of stimulation [10]. As a marker of platelet activation, MPV has attracted attention in recent decades, and many studies have evaluated its association with various malignancies [12,13,14,15,16,17,18,19,20,21,22]. The in-depth investigation of MPV alterations in cancer can reveal the potential usefulness of this index for cancer diagnosis, treatment response, and prognosis. This new perspective of a routine test may give additional information on the diagnosis and course of the disease, which is important given that several cancers may be asymptomatic until advanced stages [23].

The role of MPV in inflammatory diseases has been previously reviewed [10]. To our knowledge, there are few reviews on the relationship between MPV and cancer; including studies up to 2015, Ref. [24] is a recent review that has evaluated platelet indices with esophageal squamous cell carcinoma [25], and it includes a meta-analysis of 2421 patients, which has focused on the relation of MPV to survival in lung cancer patients [26].

Thus, the aim of this systematic review was to investigate the possible alterations of MPV in various types of cancer in relation to healthy subjects by extracting data from recent literature. In addition, the relation of MPV to disease outcome and the effectiveness of treatment was tested, and its prognostic value was assessed. Importantly, as a further goal, MPV could be used not only as a marker but also as a measure of intervention effectiveness in the disruption of tumor-platelet cross-talk.

## 2. Materials and Methods

A literature search has been made in the online databases PubMed and Google Scholar from 1 January 2010 to 31 December 2022. The review has been registered in the PROSPERO database (the University of York, https://www.crd.york.ac.uk/PROSPERO/, ID CRD42023396237, accessed on 13 March 2023).

### 2.1. Search Strategy

The following keywords were used: Mean Platelet Volume (MPV), platelet activation, cancer/tumor, gastric cancer, colon cancer, colorectal cancer, metastatic colorectal cancer, renal cell carcinoma, neoplasm, breast cancer, thyroid carcinoma, lung cancer, non-small-cell lung cancer, multiple myeloma, bladder cancer, gallbladder cancer, esophageal squamous cell carcinoma, ovarian cancer, endometrium cancer, metastasis, diagnosis, prognosis, risk factor, survival, diagnostic indicator, prognostic factors, biomarker, chemotherapy, immunotherapy, radiotherapy, and surgery.

The search terms in PubMed were formulated with Boolean operators as follows: (Mean Platelet Volume OR MPV OR platelet activation) AND (cancer OR tumor OR tumor OR carcinoma OR myeloma OR neoplasm OR survivor) AND (diagnosis OR prognosis OR risk factor OR survival OR metastasis OR diagnostic indicator OR prognostic factor OR biomarker OR chemotherapy OR immunotherapy OR radiotherapy OR surgery). We also searched the references of relative reviews for additional articles.

The research question was formulated as follows: population (P), intervention (I), comparison (C), and outcome (O). Table 1 describes in detail the research question.

### 2.2. Inclusion and Exclusion Criteria

The study selection criteria were (1) published studies within the last 12 years (publication period between 2010 and December 2022) (2) published studies in English language (3) measurement of MPV and assessment of its correlation with disease parameters or survival (4) cancer types included the following: gastric cancer, colon/colorectal cancer, esophageal squamous cell carcinoma, breast cancer, ovarian cancer, endometrial cancer, renal cancer, thyroid cancer, lung cancer, multiple myeloma, bladder cancer, and gallbladder cancer. 

Exclusion criteria were as follows: (1) studies performed on animals, (2) case reports, (3) other types of cancers not included in the inclusion criteria, (4) studies including only patients with metastatic cancer, (5) studies including only cancer survivors, (6) studies with mixed types of cancer in the study design, (7) studies assessing ratios or algorithms including MPV and not MPV alone, (8) studies assessing changes in MPV after medical treatment, (9) studies including patients with cancer and comorbidities (such as diabetes) in inclusion criteria, and (10) studies in children and/or adolescents.

### 2.3. Quality Assessment

The quality assessment of the studies is presented in Supplementary Table S1. The procedure was performed by two independent researchers (I.P. and S.P.) using the New Castle Ottawa pros scale (NOS) [27,28] for both cohort and case-control studies and the AXIS tool for cross-sectional studies [29] (Supplementary Table S2). Disagreements were discussed with a third researcher (P.D.).

### 2.4. Data Extraction

Four independent researchers (M.M., P.M., I.P., and S.P.) extracted the data in predefined excel spreadsheets with separate questions regarding MPV alterations and their relation to survival. Possible disagreements were discussed with an additional researcher (P.D.). The following items were extracted from each study: study details (first author, year of publication, design), sample details (number of participants, type of cancer, characteristics of the control group), results (increased or decreased levels in patients versus controls, outcomes about the prognostic ability of MPV, and associations with disease), and reported study limitations.

## 3. Results

**Figure 1:**
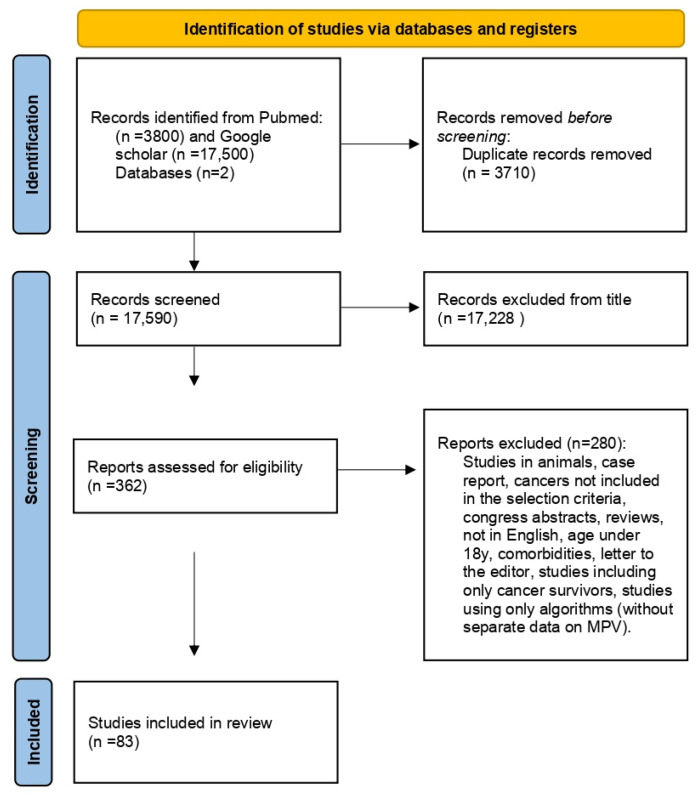
PRISMA flow diagam.

The results from the first database (PubMed) revealed 3800 articles and from the second (Google Scholar) 17,500 of the most relevant articles were read. In total, 83 studies including 21,034 participants with 12 different types of cancer, i.e., gastric cancer, colon cancer, esophageal squamous cell carcinoma, renal cancer, breast cancer, ovarian cancer, endometrial cancer, thyroid cancer, lung cancer, bladder cancer, gallbladder cancer, and multiple myeloma were identified. The flowchart according to PRISMA guidelines [30] is given in Figure 1 and the corresponding checklist is provided as a supplementary file (Supplementary Table S1) [30]. The studies are presented in detail in Table 2, Table 3, Table 4, Table 5, Table 6 and Table 7.

### 3.1. Gastric Cancer 

Eight studies including 1061 patients with gastric cancer were identified [15,31,32,33,34,35,36,37] (Table 2). The studies were conducted in China [3,35,36], Turkey [31,34] Poland [32,33], and India [37]. Most studies found that gastric cancer patients had increased MPV [3,33,34], while one found that gastric cancer patients had decreased MPV compared to controls [31]. MPV increased [37] in advanced cancer stages or remained stable [36], while surgery resulted in reductions in MPV [3,34] or no change [32]. Patients with a high [35] MPV or low [36] MPV had decreased survival, indicating an unclear relationship.

### 3.2. Colon Cancer

Regarding colon cancer, eleven studies were identified [13,19,38,39,40,41,42,43,44,45,46] including 3463 patients with colon cancer or colorectal cancer (Table 2). Seven studies were conducted in China [19,38,39,41,43,45,46], two in Turkey [40,42], one in Serbia [13], and one in Qatar [44]. Results were mixed regarding the values of MPV in patients versus controls, with one study showing increased MPV in patients with colon cancer [39] and one showing decreased MPV levels [45]. Patients with metastatic colon cancer also had increased MPV compared to nonmetastatic patients [40]. These statistically higher MPV values in metastatic disease were also found to be due to increased inflammation. The benefit of chemotherapy was also significantly greater in patients with low MPV compared with patients with high MPV [40]. Most studies found that reduced MPV is related to increased survival [38,41,42,43], while two studies had opposite findings [19,45].

### 3.3. Esophageal Squamous Cell Carcinoma

Five studies relating MPV to esophageal squamous cell carcinoma were identified, including 4258 patients [20,47,48,49,50] (Table 2). All but one [50] studies were conducted in China [20,47,48,49]. Two studies documented increased MPV in cancer patients compared to controls [49,50] and one study decreased MPV in cancer patients (pre-operatively) [20]. Results were mixed regarding the prognostic role of MPV in esophageal cancer since low MPV was related to both higher survival [47], advanced cancer stages [20], and poor prognosis [48].

### 3.4. Renal Cell Carcinoma

Regarding renal cell carcinoma, three studies were found including 681 patients from China and Poland [18,51,52] (Table 3). Patients with renal cell carcinoma had significantly reduced levels of MPV compared to patients with benign renal tumors and healthy controls [18]. Furthermore, surgical resection of the tumor led to a significant increase in MPV levels [18], and patients with low MPV had a significantly shorter survival time and worse prognosis than patients with high MPV levels [51,52].

**Table 2:** Studies relating MPV to gastric, colon, and esophageal cancer.

Reference	Study Type	Methods/Number of Participants	Results	Reported Limitations
Gastric cancer				
Aksoy et al., 2019 Turkey [31]	Case control	73 patients with gastric cancer 79 patients with intestinal metaplasia 70 healthy subjects	↓ MPV in cancer and intestinal metaplasia groups than controls.	Not mentioned
Matowicka-Karna et al., 2013 Poland [32]	Case control	13 patients with early gastric cancer (group E) 18 patients with regionally advanced cancer (group A) 19 patients with metastatic cancer (group M) 40 healthy subjects	MPV did not change after surgery (same for all groups). ↑ MPV in patients with advanced cancer.	Not mentioned
Pietrzyk et al., 2016 Poland [33]	Retrospective study- Case control	61 patients with gastric cancer 61 healthy subjects	↑ MPV in patients with cancer than controls.	Not mentioned
Kılınc¸alp et al., 2014 Turkey [34]	Retrospective study- Case control	31 patients with gastric cancer 31 healthy subjects	↑ MPV in patients with cancer than controls. No relationship between MPV and cancer stage. ↓ MPV after surgery.	Not mentioned
Shen et al., 2016 China [3]	Retrospective study- Case control	168 patients with resectable gastric cancer 30 healthy subjects (control group)	↑ Preoperative MPV in patients with gastric cancer compared with healthy controls. ↓ MPV after surgical tumor resection and ↑survival in patients, with MPV postoperative reduction.	Not mentioned
Lian et al., 2015 China [35]	Retrospective study	128 inoperable gastric cancer patients: 53 patients with locally advanced gastric tumor 75 patients with relapsed or metastatic tumor	Improved response to chemotherapy and reduced metastasis in patients with ↓ MPV level. ↑ MPV level related to ↓ survival. More favorable prognosis and better chemotherapeutic efficacy in patients with ↓MPV.	Not mentioned
An et al., 2022 China [36]	Retrospective study	401 patients who underwent gastric resection: 245 patients with stage I cancer 74 with stage II cancer 82 with stage III cancer	No significant correlation was observed between the MPV levels and clinicopathological parameters. No significant differences in MPV levels exist among gastric cancer stages. C-indices for overall survival and disease-free survival were ↑ in MPV model (age, stage, ALB, PNI, and MPV level) compared to baseline model (without MPV level). MPV was a prognostic factor for overall survival and disease-free survival, implying the clinical significance of the MPV level as a determinant of survival in gastric cancer.	Single-center retrospective study.
Manjunath et al., 2020 India [37]	Retrospective study	149 patients with gastric cancer who had chemotherapy	Significant negative association was noted between ↑ MPV and the diffuse type of histology of gastric cancer. ↑ MPV in advanced tumor stage and nodal metastases.	Retrospective study. All patients underwent chemotherapy and those with a favorable response underwent surgery which may affect the results.
Colon cancer				
Li et al., 2017 China [38]	Single-center retrospective study	509 patients with colon cancer	↓ MPV related to ↑ survival.	Single-center retrospective study. Incomplete investigation of the exact mechanism of MPV in colon cancer. Only Chinese patients.
Li et al., 2014 China [39]	Prospective study, case control	256 participants: 128 patients with colon cancer 128 healthy participants (control group)	↑ MPV in patients with colon cancer, compared to the control group.	Prospective study Lack of information about the genetic contributions to colon cancer.
Tuncel T, et al., 2014 Turkey [40]	Retrospective study	148 patients with colon cancer (53 metastatic, 95 nonmetastatic)	↑ MPV in patients with metastatic cancer, compared to nonmetastatic patients. ↓ MPV was connected to higher benefits of the treatment.	Retrospective study. Small sample number.
Liu, J. et al., 2020 China [41]	Retrospective study	873 patients with stage II-III colorectal cancer	↑ MPV is related to worse survival rates than those of patients with normal MPV levels.	Limited size of data. No external validation from other institutions.
Sakin, A, et al., 2020 Turkey [42]	Retrospective study	394 patients with Colorectal Cancer without metastasis	↑ MPV was determined as poor prognostic factor for relapse-free survival.	No inclusion of patients with metastasis. The median follow-up period was longer than that in comparable studies. Single-center retrospective study. Study did not address the mechanism of the potential effect of the MPV on prognosis of the colorectal cancer patients.
Wang, W. et al., 2021 China [46]	Retrospective study	424 patients with colorectal cancer	↓ MPV levels in microsatellite instability-high colorectal cancer patients compared with in microsatellite stable colorectal cancer patients. MPV levels were strongly associated with microsatellite instability-high status.	Lack of mechanistic explanation. Single-center retrospective study. Findings may not be generalizable to other ethnic groups.
Wang, P.et al., 2021 China [43]	Retrospective study	75 patients with locally advanced rectal cancer treated with total mesorectal excision	Patients with ↓ pre-neoadjuvant chemoradiation therapy MPV had significantly better disease-free survival.	Small number of cases. Platelet-associated could be influenced by drugs or non-tumorous diseases.
Stojkovic, Lalosevic et al., 2019 Serbia [13]	Single-center prospective study	300 newly diagnosed colon cancer patients 300 healthy volunteers (control group)	↓ MPV in patients with colon cancer.	Single-center study of newly diagnosed colon cancer patients who already had some of the symptoms.
Chang, J., Lin, G., Ye, M. et al., 2019 China [19]	Single-center retrospective clinical study	264 patients with metastatic colon cancer	Worse outcomes and reduced survival in patients with metastatic colon cancer, with ↓ MPV levels.	Single-center retrospective study. More ethnic groups need to be studied. Further explanation of the mechanism of MPV in metastatic colon cancer and the association with chemotherapy should be made.
Alsalman, A. et al., 2022 Qatar [44]	Cohort study	97 colorectal cancer patients one week prior to surgery.	↓ MPV was associated with shorter disease-free survival in left-sided colorectal cancer patients.	Relatively small sample size.
Huang L. et al., 2022 China [45]	Retrospective, case-control study	251 patients with colon cancer 171 benign colon disease cases 187 healthy controls	Healthy controls had significantly ↑MPV compared to the colon cancer cases. ↑ MPV levels in patients with stages III and IV compared to stages I and II. Correlation between MPV and the tumor size.	Relatively small sample size from a single center. Confounding factors cannot be completely ruled out. Different populations may show different levels of “inflammatory conditions.”
Esophageal squamous cell carcinoma				
Feng et al., 2019 China [47]	Retrospective study	277 resectable esophageal squamous cell carcinoma	↓ MPV related to higher cancer-specific survival in univariate analysis.	small sample size. retrospective design. exclusion of patients who received preoperative chemotherapy and/or radiotherapy. Neoadjuvant treatment will have a side effect on MPV and platelet count. Neoadjuvant treatment can improve cancer survival for locally advanced esophageal cancer but not for early-stage esophageal cancer.
Sun S-Y et al., 2018 China [20]	Retrospective single-center design	457 patients with newly diagnosed locally advanced esophageal squamous cell carcinoma who have undergone radical esophagectomy 240 healthy subjects (control group)	↓ preoperative MPV levels in patients with esophageal squamous cell carcinoma, compared to healthy control group. ↓ MPV levels in patients with advanced tumor length.	Retrospective single-center design. Lack of measurement of other inflammatory parameters such as C-reactive protein levels.
Liu X. et al., 2022 China [48]	Retrospective study	3210 patients with esophageal cancer that underwent esophagectomy	MPV served as negative prognostic factor in locally advanced-stage esophageal squamous cell cancer. ↓ MPV, as a high-risk factor, may contribute to rigorous screening for lymph node-positive staging of patients with esophageal squamous cell cancer who receive adjuvant chemotherapy.	Selection bias. Results may be affected by unit-specific practices. Did not follow up on the MPV after surgery and chemotherapy.
Zhou X., et al., 2021 China [49]	Retrospective study-case control	314 early esophageal cancer patients 329 healthy individuals (control group)	↑ MPV in early esophageal cancer patients, compared to the control group.	Using the Youden index, the sensitivities and specificities of the cut-off values were not ideal.
Feng et al., 2019 China [47]	Retrospective study	277 resectable esophageal squamous cell carcinoma	↓ MPV related to higher cancer-specific survival in univariate analysis.	small sample. retrospective design. exclusion of patients who received preoperative chemotherapy and/or radiotherapy. treatment may influence MPV.
Surucu et al., 2015 Turkey [50]	Retrospective study	52 patients with esophageal squamous cell cancer 52 with dyspepsia	↑ MPV in patients with esophageal cancer. No relation between MPV and metabolic tumor volume.	All nodal and distant metastases were not assessed. Small sample size.

**Table 3:** Studies relating MPV to renal cell carcinoma.

Reference	Study Type	Methods/Number of Participants	Results	Reported Limitations
Renal cell carcinoma				
Yun. ZY et al., 2017 China [51]	Single-center retrospective study	306 patients with renal cell carcinoma: 286 patients with locally confined disease 20 patients with locally advanced disease 290 patients with no metastasis 16 patients with metastasis	Significant shorter survival time and unexpected clinical outcomes in patients with ↓ MPV. ↓ MPV as prognostic indicator in patients with renal cell carcinoma.	Single-center retrospective. Only Chinese patients. Incomplete investigation of the exact mechanism of MPV in renal cell carcinoma.
Yun, Z., Zhang, X., et al., 2017 China [18]	Cross-sectional study	387 participants: 145 patients with renal cell carcinoma 110 patients with benign renal tumor 132 healthy control subjects (control group)	↓ MPV levels in renal cell carcinoma patients, compared with patients with benign renal tumor and healthy control group. Significant increase of MPV, after surgical tumor resection.	Cross-sectional study. Lack of information about the biochemical markers of inflammation. Study in a single center.
Prokopowicz et al., 2016 Poland [52]	Retrospective	230 patients treated for renal cell carcinoma	↓ MPV predicted cancer-specific mortality.	

### 3.5. Breast Cancer

Eight studies involving breast cancer were included with 1485 patients [16,53,54,55,56,57,58,59] from China [16,53,57], Turkey [55,56,58], Iran [59], and Greece [54] (Table 4). The extracted results indicated that the presence of breast cancer was accompanied by significantly elevated MPV levels [16,55,57,59] and metastasis development [54,55,58]. Moreover, a better response to chemotherapy was documented in patients with low MPV [56].

### 3.6. Ovarian Cancer

Six studies relating MPV to ovarian cancer were identified with 906 patients [60,61,62,63,64,65] (Table 4). Four studies were conducted in Turkey [61,63,64,65] and two studies in China [60,62]. The results were mixed since some studies documented increased levels of MPV in patients with ovarian cancer [61,65], whereas others documented decreased levels [60] or levels that were no different [62,64] from the control groups. Moreover, in the study of Kokcu, no relation was found between MPV and cancer staging [63]. It is noted that no study assessed the relation of MPV to survival in patients with ovarian cancer.

### 3.7. Endometrial Cancer

Eight studies relating MPV to endometrial cancer were identified, including 1707 patients from China and Turkey [66,67,68,69,70,71,72,73] (Table 4). In all studies, including a control group, MPV was increased in cancer versus healthy patients [66,67,69,70,71,73]. The relation of MPV with cancer staging was less clear, as in two studies, no relation was found [68,71] and in one study, MPV was negatively related to the cancer stage [67]. In two studies, MPV was tested against the overall survival with mixed results. In fact, MPV was either not related to survival [68] or was related to shorter survival (increased MPV related to lower survival) [72].

### 3.8. Thyroid Cancer

Ten studies concerning thyroid cancer were identified with 1305 participants [14,17,74,75,76,77,78,79,80,81] (Table 5). Most studies were conducted in Turkey [14,17,75,76,77,78]. The results indicated that higher MPV levels were found in patients with thyroid cancer [14,75,77,78] while one study found lower levels of MPV in cases [74] and one no difference between cases and controls [79]. Moreover, MPV was related to cardiovascular complications in patients with thyroid cancer [17] and lower MPV was related to metastasis [74,80].

### 3.9. Lung Cancer

Fifteen studies were identified regarding MPV and lung cancer with 3754 patients from China [82,83,84,85,86,87,88], Turkey [12,89,90,91], Japan [92,93], Korea [94], and Poland [95] (Table 6). Patients with lung cancer presented with higher MPV values than controls [86,87,90,91]. Several studies documented that higher MPV is related to lower survival [89,90,92,94], two had the opposite finding [12,93] and some showed no relation between MPV and survival [82,83,85,95].

**Table 4:** Studies relating MPV to breast, ovarian, and endometrial cancer.

Reference	Study Type	Methods/Number of Participants	Results	Reported Limitations
Breast cancer				
Yao et al. 2014/China [53]	Observational	608 women with breast cancer	MPV was not associated with disease-free survival.	Short mean follow-up duration. Single center. Retrospective study
Mantas et al. 2016/Greece [54]	Prospective	53 patients with early breast cancer, who developed systemic metastases over a mean follow-up period of 65 months 37 patients that remained recurrence-free	Patients with metastasis had a significantly ↑ MPV. Time to metastasis was longer in patients with low MPV.	Not mentioned.
Tanriverdi O. et al., 2016 Turkey [55]	Retrospective cohort study Case control	121 women with breast cancer and bone metastases 71 women with breast cancer without metastases (control group) 39 healthy women (control group)	↑ MPV in patients with bone metastases from breast cancer, compared to patients without metastases and the healthy control group. Correlation between high MPV with bone metastases and increased risk for inflammatory processes.	Retrospective study. Small sample size.
Mutlu H., et al., 2016 Turkey [56]	Retrospective study	109 patients with locally advanced breast cancer	Better response to chemotherapy in patients with low MPV.	Not mentioned.
Gu M. et al., 2016 China [16]	Single-center retrospective study Case control	340 women patients with newly diagnosed breast tumors: 170 women patients with invasive breast cancer 170 women with benign breast tumors (control group)	↑ MPV before therapy in patients with invasive breast cancer, compared to control group. ↑ MPV levels in patients with metastases, bigger tumor size, and tumor node metastasis stages.	Retrospective study that may not represent the whole population.
Sun H. et al., 2017 China [57]	Single-center retrospective study Case control	110 patients with breast cancer 76 healthy females (control group)	↑ MPV levels in patients with breast cancer, compared to control group. ↑ MPV in patients with lymph node metastasis and patients with high Ki67 proliferation index.	Small sample size. Retrospective study
Bozan MB., et al., 2022 Turkey [58]	Retrospective study	83 women patients with breast cancer: 46 patients with nonmetastatic axilla 37 patients with metastatic axilla	MPV was associated with development of axillary lymph node metastasis in follow up in univariable and multivariable analysis.	Single-center retrospective study. Patients were not evaluated separately in terms of their menopausal periods. Differences in the number of metastatic lymph nodes were not examined in the study.
Divsalar B., et al., 2021 Iran [59]	Retrospective study- Case control	−160 women patients with breast cancer −160 healthy controls (control group)	Moderate difference between the two groups in MPV. ↑ MPV in women with breast cancer.	Lack of follow up. No determination of the difference in hematological indices after surgery or chemotherapy and the effect of this change to predict overall survival.
Ovarian cancer				
Qin, Yuan-Yuan et al., 2018 China [60]	Retrospective study	326 patients with ovarian cancer 290 patients with benign ovarian cancer 162 healthy subjects (control group)	↓ MPV levels in patients with ovarian cancer, compared to healthy control group and patients with benign ovarian cancer.	Small retrospective study. Small sample size. Only Chinese patients.
Kemal et al., 2014 Turkey [61]	Retrospective study	113 ovarian cancer patients 90 healthy subjects (control group)	↑ MPV in preoperative cancer patients compared with healthy subjects. ↓ MPV after surgery.	Patients with heterogeneous cancer staging. Surgery failure in 2/3 of patients.
Ma et al., 2013 China [62]	Retrospective study	182 patients with epithelial ovarian cancer 122 patients with benign ovarian tumor 150 healthy women	Νο difference between patients and controls.	Retrospective study
Kokcu et al., 2014 Turkey [63]	Retrospective study	100 patients with epithelial ovarian cancer	No relation between MPV and cancer staging (I-II vs. III-IV).	Nonhomogenous population. Small sample size.
Bakacak et al., 2016 Turkey [64]	Retrospective study	185 benign cases 33 malignant cases following surgery for an initial diagnosis of adnexal mass and confirmed ovarian masses.	Νο difference between groups.	Retrospective study. Small sample size.
Yilmaz et al., 2017 Turkey [65]	Retrospective study	33 patients with ovarian cancer 33 patients with benign tumors	↑ MPV in cancer patients.	Not reported.
Endometrial cancer				
Kurtoglu, Emel et al., 2015 Turkey [66]	Retrospective study	114 patients surgically staged for endometrium adenocarcinoma (malign endometrium diseases) 105 patients who have undergone total abdominal or vaginal hysterectomy for benign uterine diseases	↑ MPV in malign endometrium diseases group, compared to patients with benign uterine diseases.	Smaller percentage of the advanced-stage endometrium cancer, compared to the early-stage group.
Zhang et al., 2020 China [67]	Retrospective	144 patients with endometrial cancer (stage I: 32; II: 42; III: 48; and IV: 22) 104 patients with endometrial hyperplasia 80 healthy subjects	↑ MPV in cancer patients compared to controls. MPV was negatively correlated with endometrial cancer staging.	Retrospective Small sample size.
Temur et al., 2018 Turkey [68]	Retrospective	763 patients with endometrial cancer	No significant correlation between MPV and endometrial cancer staging. No relation between MPV and survival.	Retrospective
Karateket et al., 2015 Turkey [69]	Retrospective	55 endometrial hyperplasia cases, 34 endometrial cancer cases 105 normal endometrial biopsy cases	↑ MPV in cancer patients.	Retrospective Small sample size.
Oge et al., 2013 Turkey [70]	Retrospective	291 patients with endometrial cancer 250 women (control group)	↑ MPV in cancer patients. ↑ MPV in advanced stages.	No data on survival status.
Song et al., 2019 China [71]	Retrospective	45 patients with endometrial cancer 143 malignant cases	↑ MPV in cancer patients. No relation between MPV and cancer staging.	Retrospective Small sample size. Small sample size for the comparison of stages III- IV to I -II group.
Chen et al., 2020 China [72]	Retrospective	1198 patients with endometrial cancer	↑ MPV was related to lymph node involvement and advanced cancer staging. ↑ MPV was associated with shorter overall survival.	Retrospective No data on tumor size.
Abide et al., 2018 Turkey [73]	Retrospective	97 patients with endometrial carcinoma 135 patients with endometrial hyperplasia 184 healthy subjects	↑ MPV in endometrial carcinoma group and endometrial hyperplasia group compared to controls.	No data on inflammatory markers.

**Table 5:** Studies relating MPV to thyroid cancer.

Reference	Study Type	Methods/Number of Participants	Results	Reported Limitations
Thyroid cancer				
Yu et al., 2017 China [74]	Cross-sectional	280 patients with thyroid cancer 280 control subjects	↓ MPV in patients with thyroid cancer. ↓ MPV related to the prevalence risk of thyroid cancer in multi-adjusted analysis MPV related to tumor-nodus-metastases stage and lymph node metastasis. Follicular carcinoma patients with T3 + T4 and lymph node metastasis had ↓ MPV in relation to the patients with T1 + T2.	Cross-sectional study. No data on genetic background.
Sit et al., 2019 Turkey [75]	Retrospective	101 patients with malignant thyroid nodules 98 patients with benign thyroid nodules	↑ MPV in the thyroid cancer group than in the benign nodule group.	Retrospective study. small sample size. sonography, cytology and scintigraphy data were not tested against MPV.
Dincel et al., 2017 Turkey [76]	Retrospective	65 papillary thyroid carcinoma patients 65 multi-nodular goiter patients 30 normal healthy subjects	No difference in MPV between groups.	Retrospective study. small sample size.
Yildiz et al., 2019 Turkey [77]	Retrospective	53 patients with papillary thyroid cancer 37 with nodular hyperplasia	↑ MPV in the thyroid cancer group with a diameter of 1 cm.	Retrospective study. small sample size.
Wen et al., 2018 China [96]	Retrospective	558 patients newly diagnosed with papillary thyroid cancer	↓ MPV was prognostic of coexistence with Hashimoto’s thyroiditis.	Retrospective study. Medication may affect results. small sample size.
Kutluturk F. et al., 2019 Turkey [17]	Retrospective study	58 patients with papillary thyroid carcinoma	↑ MPV in patients with papillary thyroid carcinoma 6 months after therapy with radioactive iodine. ↑ MPV values contributed to an ↑ risk of cardiovascular complications.	Limited patient number. Short time of follow up.
Baldane S, Ipekci SH, Sozen M, Kebapcilar L. 2015 Turkey [14]	Retrospective study	98 patients who underwent a total thyroidectomy: 66 patients with papillary thyroid cancer 32 patients with benign goiters 28 healthy subjects (control group)	↑ preoperative MPV levels in patients with papillary thyroid cancer, compared to benign goiters patients and healthy controls. ↓ MPV levels after surgical treatment of papillary thyroid cancer patients.	Retrospective study. Relatively small sample size.
Bayhan Ζ., et al., 2016 Turkey [78]	Retrospective study	146 patients who underwent total thyroidectomy: 47 patients with malignant diseases of the thyroid 99 patients with benign diseases of the thyroid	↑ MPV in patients with malignant thyroid diseases, compared to those with benign thyroid diseases.	Retrospective study. Small sample size.
Li, et al., 2022 Japan [80]	Retrospective study	212 patients with papillary thyroid carcinoma	↓ MPV was predictive of largest lymph node metastasis size ≥ 1 cm. MPV may be possible potential biomarker for evaluating the clinicopathologic features as well as prognosis of intermediate-and high-risk papillary thyroid carcinoma.	Single-center retrospective study Small sample size. Might be some other possible affective factors undetectable or predictable.
Li, C. et al., 2022 Japan [81]	Retrospective study	68 patients diagnosed with medullary thyroid carcinoma who underwent surgery	MPV was predictive of capsule invasion and postoperative Calcitonin progression. MPV potential biomarker for predicting the clinicopathological features and prognosis of medullary thyroid carcinoma.	There may have been some unknown or undetectable factors that could potentially influence the results. Number of cases relatively small.
Martin S. et al., 2021 Romania [79]	Retrospective study	265 patients diagnosed with thyroid cancer 249 patients with histologically differentiated thyroid cancer 234 papillary thyroid carcinomas 15 follicular thyroid carcinomas 108 patients with benign thyroid pathology (control group)	Papillary thyroid cancer patients had similar MPV levels when compared with the controls. Patients over 55 years old had higher MPV. Preoperative values for platelet indices (including MPV), were similar to the postoperative determinations in papillary thyroid cancer patients.	Retrospective design of the study.

**Table 6:** Studies relating MPV to lung cancer.

Reference	Study Type	Methods/Number of Participants	Results	Reported Limitations
Lung cancer				
Cui et al., 2017 China [82]	Retrospective study	270 patients with non-small-cell lung cancer	MPV was not related to survival.	Retrospective study. Single center. No mechanistic explanation provided. Study sample includes only Chinese subjects.
Hur et al., 2020 Korea [94]	Retrospective study	116 patients with non-small-cell lung cancer	↓ MPV related to low overall survival.	Some limitations reported relating to other blood indices.
Sakin et al., 2019 Turkey [89]	Retrospective study	90 patients with limited disease small-cell lung cancer	↑ MPV in patients with low total lymphocyte count. No relation between MPV and survival.	single center. no mechanism provided.
Shen et al., 2019 China [83]	Retrospective study	138 patients with non-small-cell lung cancer who underwent etoposide-based first-line chemotherapy	↑ MPV was independently related to lower survival.	Retrospective study. Single center. myelosuppression may take place due to chemotherapy drugs.
Shi et al., 2018 China [84]	Retrospective study	169 advanced and metastatic patients with non-small-cell lung cancer	No significant correlation with overall survival.	Retrospective study. Single center. Small sample size.
Wang et al., 2019 China [83]	Retrospective study	101 patients with resectable lung cancer	No significant correlation between MPV and overall survival. No effect of surgery on MPV values.	Retrospective study. Single center. Small sample size.
Watanabe et al., 2018 Japan [92]	Retrospective study	82 advanced or recurrent patients with non-small-cell lung cancer with common EGFR mutation	↑ MPV related to shorter survival. MPV was not associated with metastasis but was significantly increased in patients with smoking history.	Retrospective design. Small sample size. The used cut-off point of MPV can be applied only when using a specific analyzer Possible errors in estimating survival (high censoring).
Kumagai S., et al., 2015 Japan [93]	Retrospective study	308 patients with non-small-cell lung cancer who underwent surgery	↓ MPV levels were connected to↓ survival and poor prognosis in patients with non-small-cell lung cancer. ↓ MPV in patients with advanced non-small-cell lung cancer.	Retrospective design. Relatively short time of follow up.
Omar M., et al., 2018 Turkey [90]	Retrospective study	496 patients with non-small-cell lung cancer	↑ MPV in patients with non-small-cell lung cancer and patients with metastases. ↑ MPV was connected to ↓ survival and poor prognosis.	Relatively small sample size. Unequal distribution of histologic types.
Sakin A., Secmeler S., Arici S., et al., 2019 Turkey [12]	Retrospective study	115 patients with locally advanced non-small-cell lung cancer who received chemotherapy	Patients with low MPV had shorter survival times. Low MPV was determined as an unfavorable risk factor.	Retrospective design. Small sample size.
Kharel et al., 2022 [26]	Meta-analysis	2421 patients with lung cancer	No significant association of MPV with overall survival.	The inclusion of retrospective studies. Only English-language studies. Heterogeneity resulting from various factors may have affected the results. Univariate analyses.
Ai L., et al., 2022 China [88]	Retrospective study	703 lung adenocarcinoma patients: 270 malignant pleural effusion patients 433 tuberculous pleural effusion patients	Patients with ↑ MPV were more likely to be diagnosed as lung adenocarcinoma-associated MPE rather than TPE. MPV had strong multicollinearity and comparable diagnostic value.	Selection bias. Patients do not fully represent populations from other regions and countries. Prognostic data, such as overall survival of these patients, were unavailable.
Goksel S., et al., 2021 Turkey [91]	Retrospective, case-control study	180 patients with lung cancer 180 healthy controls	↑ MPV in patients who have not yet received any treatment and newly diagnosed lung cancer compared to healthy individuals and that as the stage progressed in patients with lung cancer diagnosis. ↑ MPV was found in lung cancer patients compared to the control group. Negative correlation between MPV and advanced disease.	Retrospective study. Number of patients at each stage is different in the distribution of patients with lung cancer.
Zhu X., et al., 2020 China [86]	Retrospective, case-control study	209 patients with lung cancer 236 healthy subjects	↑ MPV in lung cancer patients compared to healthy subjects. MPV had statistically significant correlations with neutrophil-to-lymphocyte ratio and platelet-to-lymphocyte ratio in both lung cancer patients and healthy subjects, confirming the inflammatory role of MPV in lung cancer. MPV can improve diagnostic ability to distinguish lung cancer patients from healthy subjects.	Did not divide lung cancer into small-cell lung cancer and non-small-cell lung cancer. Did not evaluate therapy measures involving surgery, chemotherapy, and radiotherapy, which could influence the levels of MPV.
Zu R., et al., 2020 China [87]	Prospective, case-control study	245 participants 159 lung cancer patients 86 normal participants	↑ MPV in patients with lung cancer.	Limitation of patient groups.
Łochowsk M., et al., 2022 Poland [95]	Retrospective study	532 patients with non-small-cell lung cancer staged IA–IIIA	The univariate analysis revealed a relationship between MPV values and patient survival. Multivariate analysis showed no significant relationship.	A single-center retrospective study. Some patients were characterized by postoperative stages IIB and IIIA and so received complementary treatment, which would have impacted the analyzed survival time.

### 3.10. Bladder Cancer

Five studies relating MPV to bladder cancer were identified with 879 participants from China [97,98,99] and Turkey [100,101] (Table 7). The results were mixed. One study documented increased levels of MPV in bladder cancer patients compared to controls [99]. Regarding recurrence risk, one study found that increased MPV was related to recurrence risk [98], while two studies found a non-significant association [100,101]. One study assessed the relation of MPV to mortality and found that lower MCV was related to lower survival [97].

### 3.11. Gallbladder Cancer

Three studies assessed the relation of MPV to gallbladder cancer, including 473 patients from China [22], Turkey [102], and India [103] (Table 7). Gallbladder cancer patients (after surgery) had lower levels of MPV than controls [22,102]. Moreover, there was no correlation between MPV and the local dissemination and prognosis of gallbladder cancer [103].

### 3.12. Multiple Myeloma

Only one study, including 62 Chinese patients with newly diagnosed multiple myeloma, was identified [104] (Table 7). This study showed significantly decreased survival in patients with low MPV, compared to patients with high MPV [104].

**Table 7:** Studies relating MPV to other types of cancer (bladder cancer, gallbladder cancer, and multiple myeloma).

Reference	Study Type	Methods/Number of Participants	Results	Reported Limitations
Bladder cancer				
Wang, Xin, et al., 2017 China [97]	Retrospective study	218 patients with bladder cancer who have undergone radical cystectomy	↓ MPV was associated with significantly decreased survival.	Retrospective study. Incomplete investigation of the exact mechanism of decreased MPV in bladder cancer. Only Chinese patients.
Song et al., 2022 China [98]	Retrospective study	271 patients after transurethral resection of bladder tumor	↑ MPV associated with recurrence.	Retrospective design. Small sample size.
Liu et al., 2019 China [99]	Retrospective study	−210 subjects with bladder cancer −76 subjects with urothelial papilloma −132 healthy control subjects	↑ MPV in bladder cancer patients and urothelial papilloma patients compared to control.	Retrospective design. Single center.
Albayrak et al., 2016 Turkey [100]	prospective study	86 patients with newly diagnosed non-muscle-invasive bladder cancer classified by the number of points assigned by the European Organization for Research and Treatment of Cancer risk tables.	No significant association between MPV progression and score, recurrence score.	Not reported.
Yildiz et al., 2021 Turkey [101]	prospective study	94 consecutive patients newly diagnosed with non-muscle-invasive bladder cancer	No significant association between MPV and disease progression nor recurrence after a follow up of 11 ± 6.4 months.	Small sample size. Single-center study. laboratory values are dependent on many factors such as nutritional deficiencies, comorbidities, medication, and lifestyle.
Gallbladder Cancer				
Zhang, Xin et al., 2018 China [22]	Cross-sectional study	213 participants: 104 patients with gallbladder cancer who had undergone surgical resection and had not received chemotherapy prior to surgery 109 healthy controls (control group)	↓ MPV levels in patients with gallbladder cancer, compared to control group.	Cross-sectional study that failed to show a causal relationship between MPV and gallbladder cancer. This study took place in only one hospital.
Kucuk, S.; Mızrak, S. 2021 Turkey [102]	Retrospective, Cross-sectional study	187 cholecystectomy specimens that were diagnosed as cholecystitis, dysplasia, and adenocarcinoma.	↑ MPV levels were found to be related to active inflammation, whereas ↓ MPV levels were found to be related to several chronic diseases. ↓ MPV in cancer and dysplasia groups.	Lacking data on the use of medication including preoperative use of anti-inflammatory therapy and the medical conditions of participants.
BV, P. et al., 2021 India [39]	Retrospective study	73 patients with gallbladder cancer	No correlation between increased MPV values and the local dissemination and prognosis of gallbladder cancer.	Based on case records, the details pertaining to each case were limited in nature. Some patients underwent chemotherapy, which may have influenced the attributes of the disease.
Multiple myeloma				
Zhuang Q. et al., 2016 China [104]	Retrospective study	62 patients with newly diagnosed multiple myeloma	Significant decreased survival in patients with low MPV, compared to patients with high MPV.	Retrospective study No complete and detailed information on the influence factors for MPV, such as smoking behavior.

## 4. Discussion

The aim of this systematic review was to investigate the alterations of MPV in various types of cancer in relation to healthy subjects and to assess the relation of MPV to disease outcomes according to data published from 2010 to 2022. In total, 83 studies with 21,034 participants and 12 different types of cancer were included, i.e., gastric cancer [15,31,32,33,34,35,36,37], colon cancer [13,19,38,39,40,41,42,43,44,45,46], esophageal squamous cell carcinoma [20,47,48,49,50], renal cancer [18,51,52], breast cancer [16,53,54,55,56,57,58,59], ovarian cancer [60,61,62,63,64,65], endometrial cancer [66,67,68,69,70,71,72,73], thyroid cancer [14,17,74,75,76,77,78,79,80,81], lung cancer [12,82,83,84,85,86,87,88,89,90,91,92,93,94,95], bladder cancer [97,98,99,100,101], gallbladder cancer [22,102,103], and multiple myeloma [104]. As can be seen, the role of MPV has been extensively investigated in several types of cancer, such as gastric cancer [15,31,32,33,34,35,36,37], colon cancer [13,19,38,39,40,41,42,43,44,45,46], breast cancer [16,53,54,55,56,57,58,59], and lung cancer, while few data exist for other types, such as renal cancer [18,51,52], gallbladder cancer [22,102,103], and multiple myeloma [104]. Most studies were conducted in China and Turkey and had a retrospective design, while the number of participants varied from 33 [65] to ~3000 [48], with an average number of participants of ~250 (mean value of all studies). The heterogeneity in research design and research questions makes comparisons between studies difficult, but several points can be highlighted.

### 4.1. Alterations of MPV Values in Patients with Cancer and Relation to Survival

An increase in MPV is observed in many neoplastic diseases, although in some cancers, a decrease can be found. More particularly, most studies in gastric (3 studies out of 4), breast (3 studies out of 3), endometrium (6 studies out of 6), thyroid (4 studies out of 6), and lung cancer (4 studies out of 4) documented an elevated MPV in cancer patients. Data regarding an increased MPV in cancer patients were less clear-cut for esophageal cancer (2 studies out of 3), ovarian cancer (2 studies out of 5), and colon cancer (1 study out of 2). In contrast, reduced MPV was observed in renal cell carcinoma (1 study out of 1) and gallbladder cancer (2 studies out of 2), although the number of studies was small (see the “Results” section). These conflicting results denote that specific organs and different types of tumors or stages of cancer may affect MPV differently or that more data are needed to obtain a clearer image.

For the case of gallbladder cancer, the observed reductions in MPV may be a result of surgery, since participants had undergone surgery at the time of measurement [22,102]. In fact, surgery seems to reduce MPV in several cancer types, such as thyroid cancer [14], ovarian cancer [61], and gastric cancer [3,34], although some studies have shown no changes [32]. This postoperative decrease in MPV values may be due to a reduced systemic inflammatory response after the reduction of tumor volume. In addition, anesthetics used in surgery may have anti-inflammatory effects [105]. For example, propofol inhibits cyclooxygenase; thus, it restricts angiogenesis, which may have an effect on MPV [106]. It is noted that other therapies, such as chemotherapy, may also reduce MPV, reflecting a reduction in inflammatory burdens [56].

MPV increased in advanced cancer stages in gastric cancer [37], esophagus cancer [20], endometrial cancer [70], and lung cancer [91]. However, other studies have not documented changes in cancer severity for gastric cancer [36] and endometrial cancer [68,71].

An increase in MPV was associated with reduced survival in most but not all studies. More particularly, most studies in colon cancer (4 out of 6) and fewer in lung cancer (4 out of 10) indicated an unfavorable role of increased MPV regarding mortality. It is noted that MPV was not related to overall survival in a recent meta-analysis of lung cancer patients [26]. As far as other cancer types are concerned, fewer studies were conducted. The available data suggest that high MPV is related to better survival in renal cancer (two out of two studies), bladder cancer (one out of one study), and multiple myeloma (one out of one study) (see the “Results” section).

**Figure 2:**
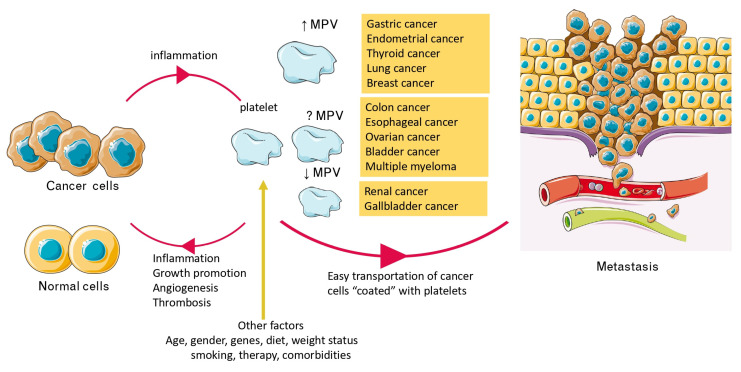
The bidirectional relation of cancer and MPV. Parts of the figure were drawn using pictures from Servier Medical Art. Servier Medical Art by Servier is licensed under a Creative Commons Attribution 3.0 Unported License (https://creativecommons.org/licenses/by/3.0/, accessed on 13 March 2023).

The relation between cancer and MPV is bidirectional and is analyzed below (Figure 2).

### 4.2. Cancer-Related Inflammation, Platelets, and MPV

Any deviations in the platelets number, total mass, morphology, and function depend on the factors that directly affect the majority of megakaryocytes, the maturity of progenitor cells, and the activation and “use” of platelets during coagulation and inflammatory processes [10]. The course of an inflammatory state is associated with an increased percentage of large platelets, possibly due to intracellular synthesis of procoagulant and proinflammatory factors, degranulation of granules, and initiation of transmigration of platelets stored in the spleen [10]. At the same time, these cells rapidly move to the site of inflammation, where they undergo activation [10].

Thus, MPV alterations in cancer patients reflect cancer-related inflammation [10]. The proinflammatory cytokines released in cancer, such as interleukins IL-1, IL-3, and IL-6, can promote the proliferation of megakaryocytes and increase the presence of large platelets, causing their activation and aggregation and possibly leading to the gradual establishment of thrombocytosis [35]. In parallel, higher IL-6 levels are associated with increasing tumor stages, tumor sizes, metastasis, and reduced cancer survival [10,39].

Moreover, for stomach cancer, a chronic inflammation caused by Helicobacter pylori may be present, which is often leading to neoplastic transformation [3,13]. On the contrary, in some cancers, for example, renal cancer, a decrease in MPV was observed [18]. In this case, it can be hypothesized that the inflammatory state accompanying carcinoma may lead to excessive “usage” of platelets and consequently a decrease in MPV, which is reversed upon anti-inflammatory treatment [10,18,51].

### 4.3. MPV, Activated Platelets, Cancer Progression, and Metastasis

Platelets have an important metabolic role in cancer pathogenesis through their angiogenic, metastatic, and proteolytic activities in the context of inflammation [4,10]. Activated platelets facilitate cancer progression and tumor growth by promoting angiogenesis and tumor cell generation at distant sites through the secretion of angiogenic growth factors, such as the vascular endothelial growth factor (VEGF) [4,10,107]. Indeed, the platelet content of VEGF is significantly increased in cancer patients [108].

Thrombosis is one of the common causes of mortality in cancer patients and the clotting process is enhanced by activated platelets through their procoagulant surface [109]. Multifactorial complex interactions between platelets, endothelial cells, and leukocytes further stimulate the production of proinflammatory cytokines and lead to thrombosis [109]. Recent studies have provided ample evidence for the multifunctional nature of platelets, which are the first to accumulate at the site of injury, changing shape and exhibiting pseudopodia and local release of cytoplasmic granular contents [109]. When activated by classical agonists such as ADP, TXA2, PAF, inflammatory cytokines (e.g., IL-1, IL-6), tumor necrosis factor alpha (TNF alpha), and other growth, hemostatic factors and the adhesion molecules they aggregate [7,8] stimulate platelet production and lead to a hypercoagulable state and thrombogenesis [4,10].

Platelets also play an important role in cancer progression and metastasis [110]. Elevated thrombocytosis and platelet count are associated with advanced, often metastatic, stages of cancer as also supported in our results for colon and breast cancer [40,54,55,58], but not for thyroid cancer [80]. Activated platelets create a procoagulant microenvironment that allows cancer cells to become coated with platelets and evade the host’s immune system. Encrusted with platelets, circulating cancer cells can more easily transport themselves into the bloodstream and cope with physical factors, such as shear stress when passing through the microvascular system [110]. Further in vivo studies have shown that platelets in cancer patients may mask cancer cells, making them unrecognizable by immune system cells, which facilitates metastasis [110].

Regarding the relationship between MPV and tumor aggressiveness, it is important to mention that the increased reactivity of larger platelets is due, among other things, to increased expression of integrin αIIbβ3 and glycoprotein (GP) Ibα [111]. αIIbβ3 may participate in platelet–tumor cell interaction in tumor metastasis through the binding of metalloproteinase domain-containing protein 9 (ADAM-9). This interaction could form a physical shield around cancer cells protecting them from natural killer (NK) cell lysis [112]. Additionally, it has been observed that activated platelets facilitate tumor cell adhesion to endothelial cells through αIIbβ3-associated mechanisms [113]. Moreover, activated platelets can facilitate tumor cell extravasation after the extracellular matrix degradation by matrix metalloproteinase-2 (MMP-2) [114]. Regarding the GPIba, GPIbα-mediated platelet adhesion to angiogenic vessels enhances angiogenesis and prevents hemorrhage from newly formed vessels contributing to cancer development and aggressiveness [115].

### 4.4. Other Factors Affecting MPV

Preanalytical factors may interfere in MPV determination, such as the anticoagulant used (ethylenediamine tetraacetic acid—EDTA or citrate with EDTA changing platelet shape), sample temperature (with high temperature leading to increases in MPV), and the interval between blood taking and testing [10,116].

Another issue that needs to be considered is the definition of a normal MPV range in healthy subjects, as the available literature presents different cut-off points depending on the method and the hematological analyzer used [117]. This clearly shows the need to establish reference values for MPV by laboratories and possibly a reference range of values in relation to gender, age, or ethnicity. Finally, the baseline values and differences of MPV in benign and malignant tumors, which have not yet been elucidated, also need to be determined [10,24].

Some researchers indicate that MPV should always be evaluated together with platelet count, as there is a non-linear inverse relationship between these blood indices [10]. It has been documented this ratio can have a high prognostic value in cancer patients [118]. To date, the effect of platelet count on MPV has not been fully understood in malignant tumors and the present review excluded studies using ratios of blood indices while not reporting results for MPV alone.

The value of MPV and other platelet indices can be affected by many factors, such as age [119], gender [120], and genetic factors [121]. Age was positively related to MPV in thyroid cancer patients [79]. In addition, age has been associated with tumor progression and recurrence risk and may modify the relation of blood indices (such as the neutrophil-to-lymphocyte ratio) to the disease [100]. Lifestyle (including diet and weight status) and genetic factors may also affect MPV [122,123]. So lifestyle and its improvement may play a role in reducing platelet activation and may be an aspect of treatment in some patients [10]. Moreover, cancer treatment, such as hormone therapy [124], and Radioiodine Therapy [125] may alter MPV values. Concerning antithrombotic drugs, it has been shown that aspirin does not affect MPV, but no data are available on the possible effect of other antiplatelet drugs on MPV values [10].

Various conditions and diseases, such as diabetes mellitus, hypertension, hypercholesterolemia, smoking, and obesity, show higher levels of MPV [126,127]. Smoking also seems to increase MPV in cancer patients [92]. In addition, a high level of MPV may also be associated with various malignant tumors, as already mentioned [24]. It is noted that in the studies analyzed in this review, patients with comorbidities were excluded, which is better for the interpretation of the results.

### 4.5. Limitations

Most included studies were retrospective and had a small sample size. The majority of studies were conducted in China and Turkey, which may limit the generalization of the observed findings. In the methodology of the present review, we included most types of cancer but not all, due to large data management. We also excluded the articles which used only combinations of biochemical indices to predict overall survival, since the aim of the present review was to clarify the role of MPV in cancer. However, in real life, it is possible that combinations of indices and resulting algorithms could predict an outcome better. For example, cardiovascular risk is better predicted through several algorithms combining age, sex, smoking, lipid, and blood pressure measurements [128].

In our work, we did not provide quantitative information on MPV levels in the different studies. Different cutoffs were used by researchers and thus it is difficult to define an optimal “prognostic” value of MPV within the normal range. Yet, there are no standardized reference values [116]. We documented several alterations in MPV values in cancer patients compared to a “control” group. It is noted that in some studies, the “control” group included adults with benign tumors and not healthy adults [16,78], which may have affected the magnitude and/or orientation of comparisons.

In treated patients with cancer, the timing of MPV measurement in relation to treatment (such as surgery or chemotherapy) may be also important, which is not always available. Moreover, several variables that influence MPV, such as genetic polymorphisms, body composition, and diet, were not assessed, and these may affect both cancer prognosis [129] and platelet secretory molecules [130,131,132]. In addition, usual treatments may also induce changes in biological parameters [133]. Last but not least, cancer patients often take vitamins and oral nutritional supplements to cover their nutritional needs, which most often contain fat to increase caloric supply [134]. Interestingly, MPV has been correlated with platelet unsaturated phospholipids [135].

## 5. Conclusions

Over the last few years, several studies reviewed in the present work have investigated the association between MPV and cancer in terms of diagnosis and prognosis. MPV can be used as a potential biomarker in cancer diagnosis and could be a useful tool for the optimization of treatment strategies. However, further studies are needed to elucidate the exact role of MPV in cancer progression and responsible underlying mechanisms.

## Figures and Tables

**Table 1:** Formulation of the research question (Population, Intervention, Comparison, Outcome).

P-Opulation
Patients with cancer
I-ntervention
Surgery, immunotherapy, radiotherapy, Chemotherapy
C-Comparison
Comparison of MPV in healthy and cancer patients
O-utcome
Relation of MPV to survival, severity of disease, metastasis Discriminatory and diagnostic capacity of MPV

## Supplementary Materials

The following supporting information can be downloaded at: https://www.mdpi.com/article/10.3390/curroncol30030258/s1, Table S1: PRISMA checklist. Supplementary Table S2: Quality assessment of the included studies.
